# FEN1 Promotes Hepatocellular Carcinoma Progression by Activating Cell Cycle Transition from G2 To M Phase

**DOI:** 10.7150/jca.88160

**Published:** 2024-01-01

**Authors:** Rangrang Wang, Haijiao Zhang, Dan Huang, Junming Xu, Yang Zhang, Tao Wang

**Affiliations:** 1 Huadong Hospital Affiliated to Fudan University, 221 West Yan'an Road, Shanghai, China.; 2 Department of General Surgery, Shanghai General Hospital, Shanghai Jiao Tong University School of Medicine, Shanghai, China.; 3 Digestive Endoscopic Center, Shanghai Sixth People's Hospital Affiliated to Shanghai Jiao Tong University School of Medicine, Shanghai, China.

**Keywords:** Flap endonuclease 1, Hepatocellular carcinoma, Cell cycle, G2/M transition

## Abstract

Flap endonuclease 1 (FEN1) is a structure-specific nuclease that is involved in the occurrence and development of various types of tumors. Previous studies have shown that FEN1 plays an important role in the development of hepatocellular carcinoma, however, the molecular mechanisms remain fully elucidated, especially its effect on the cell cycle of hepatocellular carcinoma has not been investigated.

In this study, via bioinformatics prediction and clinical specimen verification, we confirmed that FEN1 was highly expressed in HCC and correlated with poor prognosis. The knockdown or overexpression of FEN1 could inhibit or promote the proliferation and invasion of HCC cells. Importantly, cell cycle and functional experiments showed that FEN1 could promote cell proliferation by inducing cell cycle transition from G2 to M phase. Further studies indicated that FEN1 regulated the G2/M transition by modulating cell division cycle 25C (Cdc25C), cyclin-dependent kinase 1 (CDK1) and Cyclin B1 expressions. To sum up, our research suggested that FEN1 could promote the proliferation, migration and invasion of HCC cells via activating cell cycle progression from G2 to M phase, indicating that FEN1 may be a potential target for the treatment of HCC.

## Introduction

Hepatocellular carcinoma (HCC) is the sixth most common cancer and ranks as the third leading cause of tumor-related death worldwide 1. Although surgery is the best way to treat HCC 2, due to the insidious onset of HCC and early metastasis, the majority of patients are already in advanced stages when diagnosed and are no longer eligible for surgery 3. In recent years, the development of immunotherapy and targeted therapy has brought new hope for advanced HCC 4. Therefore, it is of great importance to find novel therapeutic biomarkers and targets.

FEN1, located on human chromosome 11q12-13, is a structure-specific nuclease involved in DNA replication, synthesis, damage repair, nonhomologous end-joining and homologous recombination 5-7. In this regard, FEN1 is essential for the maintenance of genomic stability 8, 9. Previous studies have shown that FEN1 is abnormally expressed in lung, breast, gastric, prostate and other types of cancer and is closely correlated with the occurrence and development of tumors 10-12. It has been reported that FEN1 is highly expressed in liver cancer 13. FEN1 is also highly expressed in HCC and can promote HCC progression through methylation, ubiquitination, and action on miRNAs 13-15, but its role in the HCC cell cycle remains unknown.

In view of the role of FEN1 in DNA replication, we speculated that FEN1 is crucial for the proliferation of HCC cells. In this study, we conducted bioinformatics prediction and clinical specimen verification, which confirmed that FEN1 was highly expressed in HCC and correlated with poor prognosis. We found that FEN1 could promote the proliferation, colony formation, wound healing, migration, and invasion of HCC cells. Gene set enrichment analysis (GSEA) revealed that high expression of FEN1 was significantly related to the cell cycle pathway. Cellular experiments and molecular experiments demonstrated that FEN1 regulates the cell cycle transition from G2 to M phase by modulating Cdc25C, CDK1 and Cyclin B1, thus promoting the proliferation of HCC cells. Our study suggests that FEN1 may be a potential target for the treatment of HCC.

## Material and methods

### HCC Datasets

TCGA-LIHC and corresponding clinical data used in this study were downloaded from The Cancer Genome Atlas (TCGA) portal (https://gdc-portal.nci.nih.gov/).

### Clinical specimen collection

The HCC and adjacent normal tissues analyzed in this study were collected from patients at Shanghai General Hospital between January 2013 and December 2015. Inclusion: (1) age >18 years; (2) primary liver cancer; (3) no preoperative treatment such as immunotherapy, chemotherapy, or radiotherapy; exclusion of any T, N, or M staging unknown. These patients did not receive immunotherapy, chemotherapy or radiotherapy before surgery. This research was approved by the Ethics Committee of Shanghai General Hospital and informed consent was obtained from all patients enrolled in the study.

### Real-time quantitative PCR (RT-qPCR)

According to the manufacturer's instructions, TRIzol (Takara Biotechnology, Japan) was used to extract total RNA from the tissue samples and HCC cell lines. Then, we synthesized cDNA using a reverse transcription kit (Takara Biotechnology, Japan) for subsequent PCR assay. The relative mRNA expression levels were normalized to GAPDH and calculated by the 2^-ΔΔct^ method. All samples were analyzed in three replicates. The primers are shown in Table S1.

### Immunoblot analysis

Total protein was extracted from the tissue samples and cells, and the protein concentrations were quantified using a BCA kit (Yeasen, Shanghai, China). Equivalent proteins were separated by SDS-PAGE and transferred to PVDF membranes (Millipore, Billerica, MA, USA). Then, the membranes were blocked and incubated with primary and secondary antibodies. We used ECL chemiluminescence to detect protein signals. GAPDH was used as the internal reference protein. Antibodies against the following proteins were used: GAPDH (60004-1-Ig, Proteintech), FEN1 (ab133311, Abcam), Cdc25C (ab32444, Abcam), CDK1 (ab133327, Abcam) and Cyclin B1 (ab32053, Abcam).

### Immunohistochemistry (IHC)

The sections were baked at 56°C for 2 h for dewaxing, boiled in citrate buffer for antigen retrieval, and blocked using 3% hydrogen peroxide. The contents were incubated with the primary antibody against FEN1 (1: 200, ab133311, Abcam) at 4°C overnight and biotinylated with a goat anti-rabbit secondary antibody for 1 h. At last, the reaction was visualized using DAB, and the sections were counterstained with hematoxylin. IHC scores were calculated by multiplying the percentage and intensity score of stained cells (staining intensity: negative = 0, weak = 1, moderate = 2, strong = 3, and staining extent: 0 = no staining, 1 = 0%-25%, 2 = 25%-50%, 3 = 50%-75% and 4 = 75%-100%). The total score was calculated as intensity score × extent score. Scores of > 4 were regarded as having high FEN1 expression while those with 1, 2, 3 and 4 were regarded as having low FEN1 expression.

### Cell culture and transient transfection

HCC cell lines (Huh-7, Hep-3B, Hep-G2, Bel-7402, SMMC-7721 and HCCLM3) were cultured in a humidified incubator containing 5% CO_2_ at 37°C. Lentivirus was produced by transfection of HEK-293T cells with psPAX2 and pMD2.G plasmids using Lipofectamine 2000 (Invitrogen, CA, USA) according to the manufacturer's instructions. The sequences of shRNAs are shown in Table S2.

### CCK-8 assay

Cell viability was measured using the Cell Counting Kit-8 (CCK-8) assay (NCM Biotech, Suzhou, China) to evaluate cell proliferation. Cells were seeded into 96-well plates at 2000 cells per well. The absorbance at 450nm was measured with a spectrophotometer at different time points (0, 12, 24, 48, and 72 h).

### Colony formation assay

Cells were seeded into 6-well plates at 1000 cells per well. After 2 weeks of culture, the cells were fixed and stained with crystal violet.

### Scratch wound healing assay

Cells were seeded into 6-well plates and cultured to 85% confluence. The cell layers were scratched using a sterile 200μL pipette tip and then washed three times with PBS to remove the scratched cells. Then, the remaining cells were cultured in serum-free DMEM (Gibco, USA). Cells were observed using an inverted microscope and photographed at 0 and 48 h respectively.

### Transwell assay

Transwell assay was conducted for cell migration and invasion studies. Cells in serum-free medium were seeded into the upper chamber, and 600μL DMEM containing 10% FBS (Gibco, USA) was added to the lower chamber. Matrigel (Corning, NY, USA) was used to precoat the upper chamber before cell seeding. The cells were fixed after 24 h of culture. Cells on the underside of the membrane were stained with 0.1% crystal violet and counted under a microscope.

### Flow cytometry

A cell cycle detection kit (MultiSciences, Hangzhou, China) was used to assess the cell cycle of HCC cells according to the manufacturer's instructions.

### EdU assay

Detection of EdU was conducted with the Cell-Light EdU Apollo 567 (catalog no. C10310-1; RiboBio) according to the manufacturer's instructions.

### Statistical analysis

The relationships between FEN1 expression and clinicopathological features of HCC patients were analyzed by chi-square test. Kaplan-Meier method with log-rank test was used for survival analysis. The boundary value of FEN1 expression was determined by its mean value. The data are presented as the mean ± SD. Student's t-test was used to compare two groups, and one-way ANOVA was used for comparison among multiple groups. P < 0.05 indicated a significant difference.

## Results

### FEN1 is upregulated in HCC and correlated with poor prognosis

**Figure 1:**
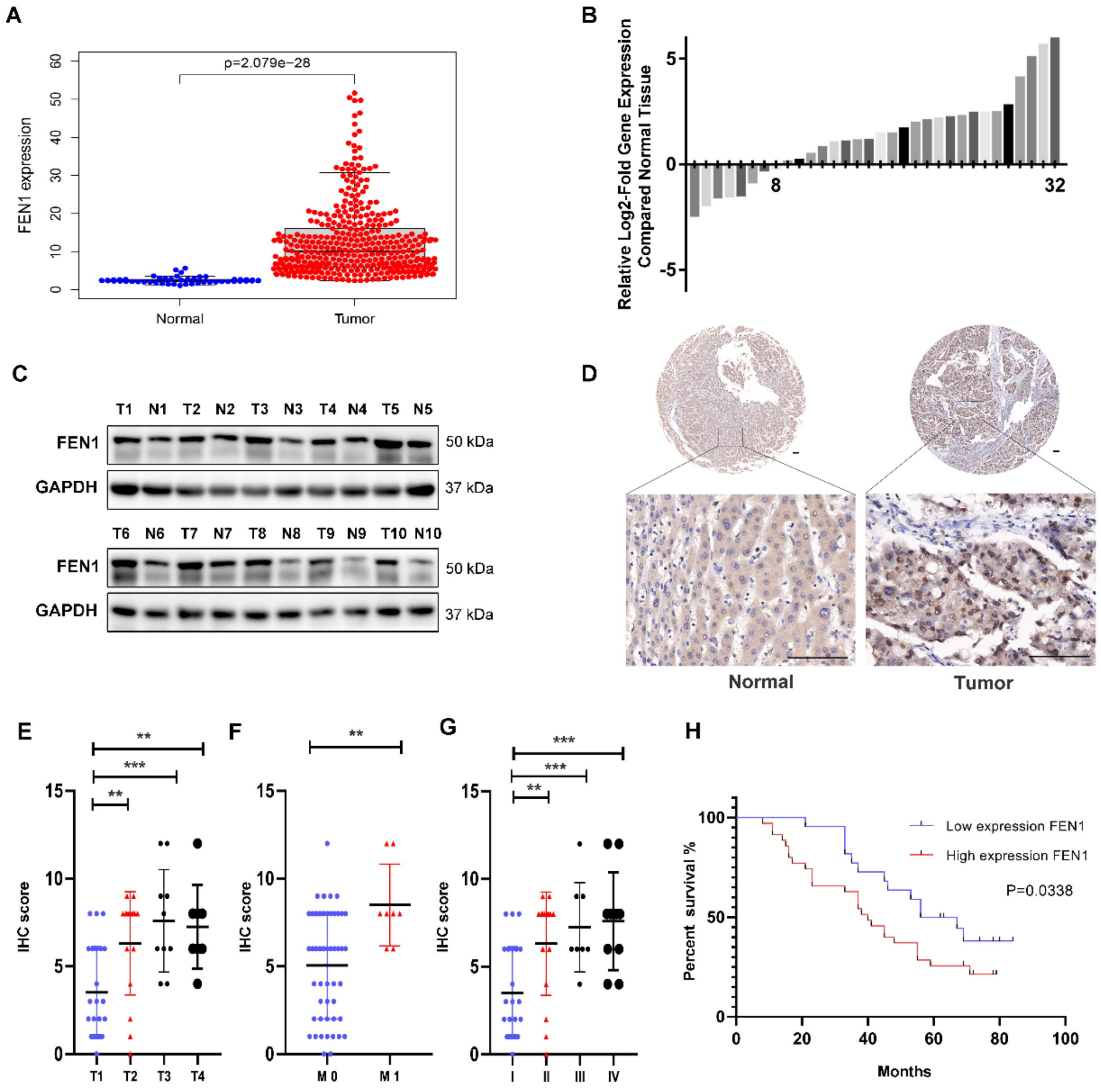
** FEN1 is upregulated in HCC and associated with clinical features.** (A) mRNA expression level of FEN1 in 374 HCC tissues and 50 normal tissues from the TCGA database. (B) RT-qPCR analysis of FEN1 expression in 32 paired HCC tissues and adjacent normal tissues. (C) Western blot detection of FEN1 protein expression in 10 representative pairs of HCC tissues and adjacent nontumor tissues. (D) IHC staining scores of FEN1 expression in 57 paired HCC tissues and adjacent normal tissues. Representative images of different FEN1 expression levels are shown. Scale bar 100μm. (E, F, G) High FEN1 expression was correlated with tumor T stage (E). tumor M stage (F) and tumor stage (G). (H) Comparison between low and high FEN1-expression groups in tissue microarray (TMA) revealed that high FEN1 expression was associated with poor overall survival. ** P<0.01, *** P < 0.001.

To explore the expression levels of FEN1 in HCC, we first downloaded the gene expression data of HCC tissues from the TCGA database and analyzed the mRNA expression level of FEN1. The results showed that FEN1 was upregulated in HCC tissues compared with normal tissues (Figure 1A). Analysis of the mRNA data in the Oncomine database yielded similar results (Figure S1A). Paired comparisons showed that FEN1 was highly expressed in HCC tissue compared with adjacent normal tissues from the TCGA database (Figure S1B).

Next, we used qPCR to analyze FEN1 mRNA levels in 32 pairs of HCC and matched adjacent normal tissues from Shanghai General Hospital. The results showed that 24/32 (75%) of the HCC tissues exhibited higher FEN1 mRNA levels than the corresponding adjacent normal tissues (Figure 1B). In addition, the results of Western blot showed that the protein expression levels of FEN1 were also upregulated in HCC tissues compared with adjacent tissues (Figure 1C). Moreover, IHC staining analysis was conducted to determine FEN1 protein expression in tissue microarray (TMA) containing 57 pairs of HCC and matched adjacent normal tissues. The protein expression levels of FEN1 were higher in HCC tissues compared with adjacent tissues (Figure 1D and Figure S1C). In summary, these findings indicated that FEN1 was upregulated in HCC tissues at both the gene and protein levels.

In addition, we analyzed the correlations between FEN1 protein expression and HCC clinicopathological features. 57 HCC patients were divided into FEN1 high-expression (61.4%, 35/57) and low-expression (38.6%, 22/57) groups according to IHC score. We found that high expression of FEN1 was positively correlated with tumor T stage, tumor M stage, tumor stage and tumor grade (Figure 1E-G and Figure S1D), while there was no correlation between FEN1 expression and gender, age or tumor N stage (Table 1). In addition, Kaplan-Meier survival analysis revealed that HCC patients with high FEN1 expression had worse prognosis than those with low FEN1 expression (Figure 1H). Analysis of the public data from the TCGA database also showed similar results (Figure S1E).

### FEN1 promotes the proliferation, migration and invasion of HCC cells

**Figure 2:**
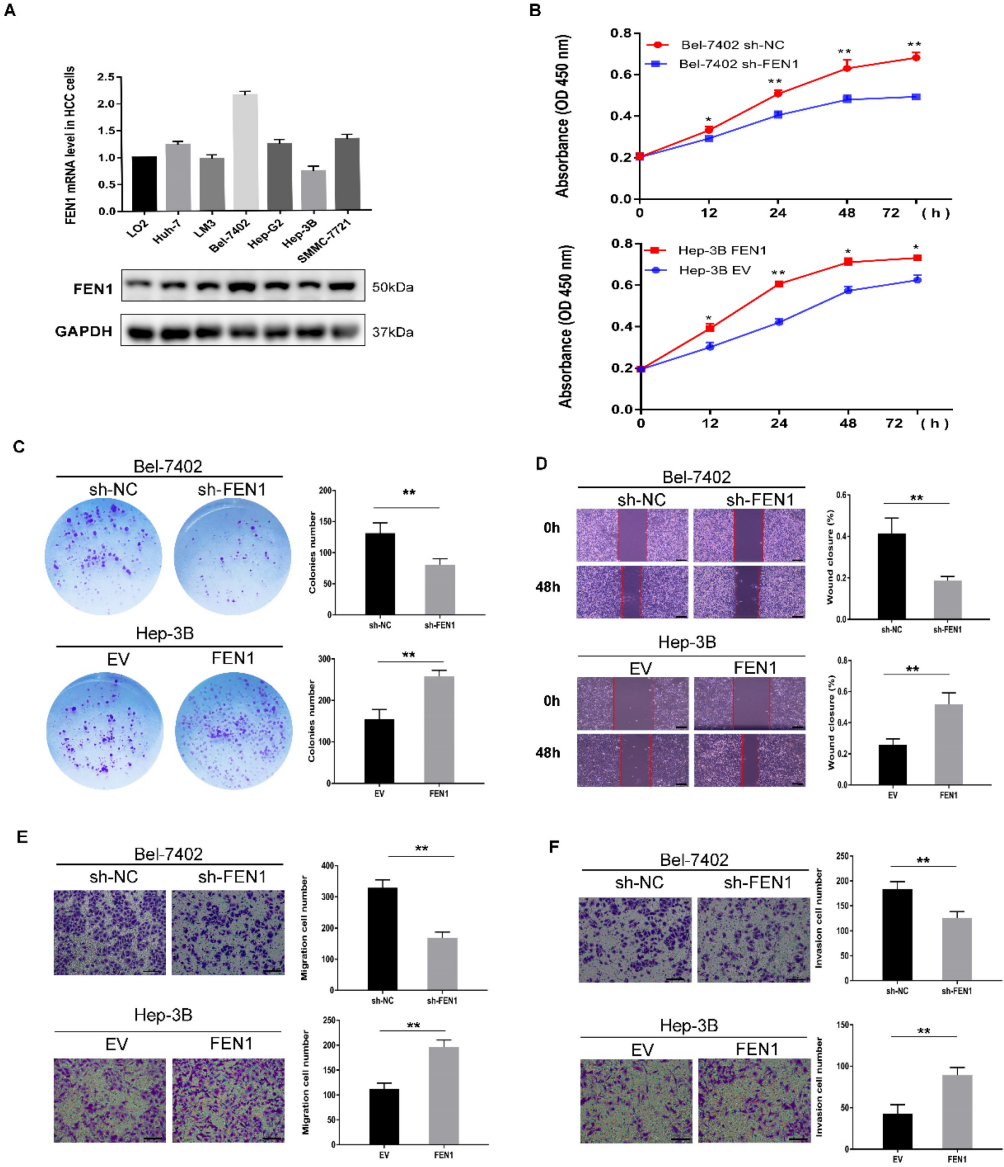
** FEN1 promotes the proliferation, migration and invasion of HCC cells.** (A) FEN1 expression in a human normal liver LO2 cell and 6 HCC cell lines. (B) CCK-8 assay revealed that knockdown of FEN1 in Bel-7402 cells significantly lowered the cell viability compared with the levels in sh-NC, whereas overexpression of FEN1 increased Hep-3B cell viability. (C) Colony formation assays revealed the effect of FEN1 expression level on proliferation ability in HCC cells. (D) Wound healing assays revealed the migration of HCC cells, and the percentage wound closure was calculated. Scale bar 200μm. (E, F) Transwell assay indicated that FEN1 knockdown inhibited the cell migration (E) and invasion (F) ability. In contrast, overexpression of FEN1 significantly enhanced the migration (E) and invasion (F) abilities of HCC cells. Scale bar 200μm. Error bars indicate the standard deviation of triplicates. * P < 0.05, ** P < 0.01.

We used qPCR and Western blot to detect the expression of FEN1 in HCC cell lines. Among the six HCC cell lines, Bel-7402 and Hep-3B cells showed the highest and lowest FEN1 expression levels (Figure 2A). Therefore, we selected Bel-7402 and Hep-3B cell lines for further experiments and established cell models of FEN1 knockdown (Bel-7402) and overexpression (Hep-3B) (Figure S2A-B). The results of CCK-8 showed that the knockdown of FEN1 in Bel-7402 cells significantly reduced cell viability compared with the levels observed in the control group, while overexpression of FEN1 increased Hep-3B cell viability (Figure 2B). Colony formation assay showed that the number of colonies was decreased significantly under FEN1 knockdown. In contrast, Hep-3B cells overexpressing FEN1 yielded the opposite results (Figure 2C). In addition, the wound healing capability of sh-FEN1 cells was significantly weaker at 48 h post-scratch compared to the control group. On the contrary, cells overexpressing FEN1 displayed significantly enhanced wound healing ability (Figure 2D). Regarding cell migration and invasion, Transwell assays showed that FEN1 knockdown markedly reduced the migration and invasion abilities of Bel-7402 cells. Conversely, overexpression of FEN1 in Hep-3B cells significantly enhanced the migration and invasion potentials of the cells (Figure 2E-F).

### FEN1 promotes the proliferation of HCC cells through activating cell cycle progression from G2 to M phase

**Figure 3:**
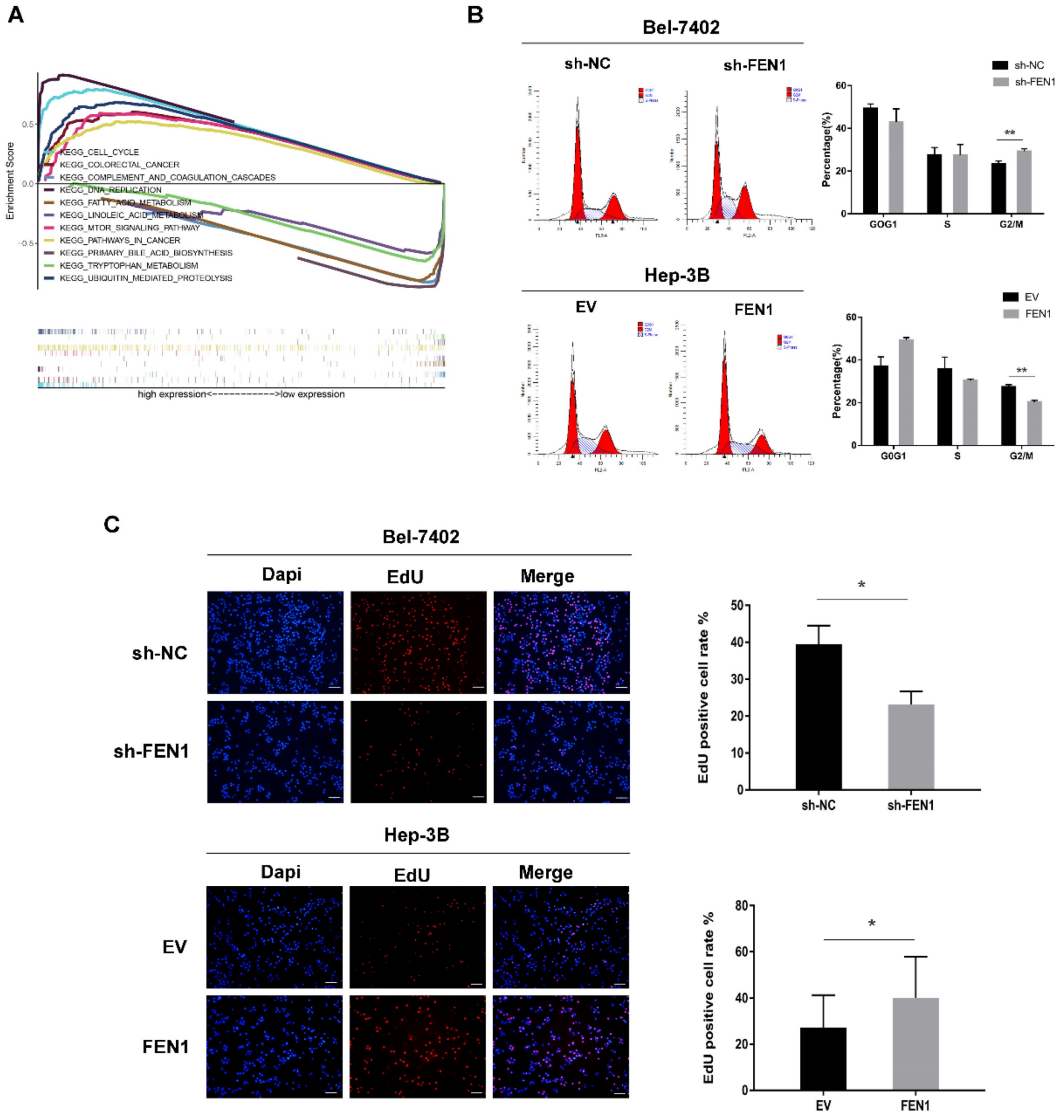
** FEN1 is involved in cell cycle G2-to-M transition.** (A) Multiple enrichment plots from gene set enrichment analysis (GSEA). (B) Representative analysis of flow cytometry for cell cycle detection, histogram of the proportions of cells in G1, S, and G2/M phases of the cell cycle. (C) EdU assay revealed the effect of FEN1 expression level on proliferation ability in HCC cells. Scale bar 200μm. Error bars indicate the standard deviation of triplicates. * P < 0.05, ** P < 0.01.

To further explore how FEN1 promotes HCC cell proliferation, GSEA of FEN1 high- and low-expression patient groups from the TCGA database was carried out. We selected the most significantly enriched pathway according to the normalized enrichment score (NES). The results showed a significant enrichment of cell cycle pathways in the patient group with the FEN1 high-expression phenotype (Figure 3A).

Subsequently, we analyzed cell cycle progression in Bel-7402 and Hep-3B cells by fluorescence-activated cell sorting (FACS). As shown in Figure 3B, FEN1 knockdown resulted in a marked increase in the percentage of G2/M-phase cells, whereas the percentage of G1-phase cells was decreased. In contrast, FEN1 overexpression resulted in a decrease in the percentage of G2/M-phase cells but an increase in the percentage of G1-phase cells. Furthermore, EdU assay demonstrated that FEN1 knockdown inhibited the proliferation of Bel-7402 cells, while FEN1 overexpression promoted the proliferation of Hep-3B cells (Figure 3C). These results suggest that FEN1 may promote HCC cell proliferation by activating cell cycle progression from G2 to M phase.

### FEN1 regulates the cell cycle transition from G2 to M phase by modulating Cdc25C, CDK1 and Cyclin B1 expressions

**Figure 4:**
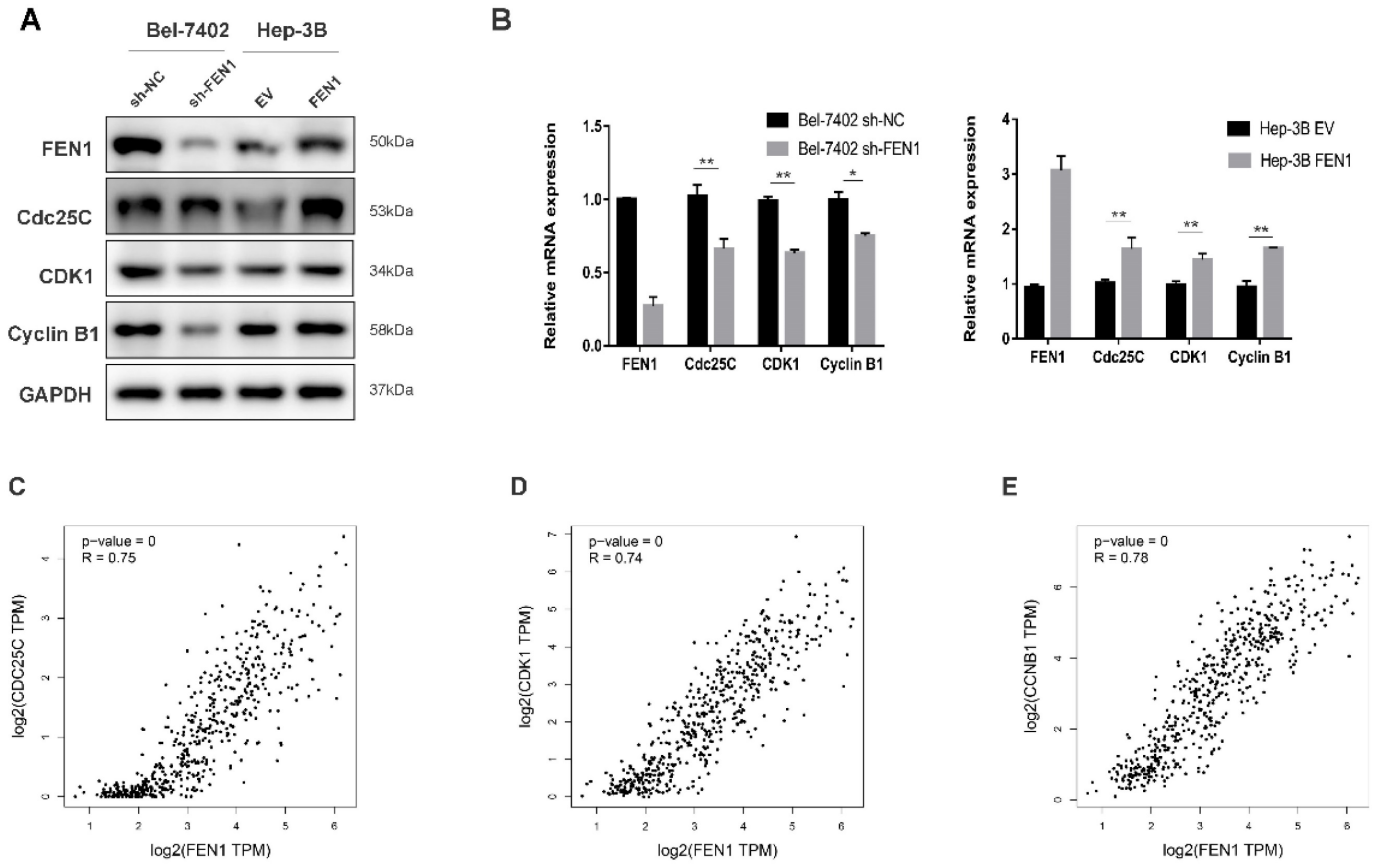
** FEN1 regulates the cell cycle transition from G2 to M phase by modulating Cdc25C, CDK1 and Cyclin B1 expressions.** (A) Western blot demonstrated that the knockdown of FEN1 decreased the protein levels of Cdc25C, CDK1 and Cyclin B1 in Bel-7402 cells. Conversely, overexpression of FEN1 increased the levels of these proteins in Hep-3B cells. (B) The mRNA levels of FEN1, Cdc25C, CDK1 and Cyclin B1 in Bel-7402 and Hep-3B cells, GAPDH was used as the internal control, and the experiment was repeated at least three times independently. (C, D, E) FEN1 expression was positively correlated with Cdc25C, CDK1 and Cyclin B1 expressions. Spearman coefficient R=0.82, 0.75 and 0.74 respectively; P < 0.001. * P < 0.05, ** P < 0.01.

To investigate the underlying mechanism of the proliferation-promoting function of FEN1, cell cycle-related proteins were analyzed using Western blot and RT-qPCR. The Cyclin B1/CDK1 complex is crucial to regulating G2 transition 16. Moreover, Cdc25C can activate the Cyclin B1/CDK1 complex by inducing CDK1 dephosphorylation to promote mitotic cell G2/M transition 17. Western blot demonstrated that the knockdown of FEN1 decreased the protein level of Cdc25C in Bel-7402 cells, and the expression levels of CDK1 and Cyclin B1, the downstream proteins of Cdc25C, were also decreased under FEN1 knockdown. Conversely, overexpression of FEN1 increased the level of Cdc25C, CDK1 and Cyclin B1 in Hep-3B cells (Figure 4A). The RT-qPCR results showed similar alterations to the protein expressions (Figure 4B). In addition, we analyzed gene expression correlations between FEN1 and Cdc25C, CDK1 or Cyclin B1 using a bioinformatics tool, GEPIA (http://gepia.cancer-pku.cn/). The results showed that FEN1 mRNA level was positively correlated with the mRNA expression levels of Cdc25C, CDK1 and Cyclin B1 (Figure 4C-E). In summary, our data suggest that FEN1 may promote cell cycle transition from G2 to M phase by modulating Cdc25C, CDK1 and Cyclin B1 expressions, thus promoting the proliferation of HCC cells.

## Discussion

As the third leading cause of cancer-related deaths in the world, liver cancer has brought a heavy cancer burden to many countries 16. Given the low detection rate of early-stage liver cancer, metastasis and intraperitoneal spread have already occurred at the time of diagnosis in the majority of patients; the overall 5-year survival rate for liver cancer patients is only about 20% 17, with poor prognosis.

The occurrence and development of liver cancer is a complex biological process involving the interaction of multiple molecules that are regulated by key genes 18, 19. The research on the regulatory mechanism of key genes and the progress of molecularly targeted drugs provides new hope for the treatment of liver cancer 20, 21. As a structure-specific 5'-nuclease, FEN1 plays important roles in DNA replication and damage repair 22. In addition, studies have shown that FEN1 is highly expressed in various types of cancer cells and is closely associated with the occurrence and development of tumors 10, 12. These findings suggest that FEN1 may act as a double-edged sword in cancer.

Our study showed that FEN1 was significantly upregulated in HCC and that high FEN1 expression was associated with higher tumor T stage, tumor M stage and tumor stage. Moreover, K-M analysis revealed that high expression of FEN1 is indicative of poor prognosis in HCC patients, consistent with the findings of Li et al 13. Next, we revealed that the knockdown of FEN1 inhibited the proliferation, migration and invasion of HCC cells, whereas the overexpression of FEN1 promoted cell proliferation, migration and invasion, indicating that FEN1 plays a vital role in the development of HCC.

Cell cycling is a complex process involving a series of cell cycle regulators 23, 24. At different times, different cell cycle regulators have different expression and degradation patterns, culminating in the division of a mother cell into two daughter cells through mitosis 25, 26. In this study, we found through GSEA analysis that high expression of FEN1 was closely associated with the cell cycle. In addition, cell cycle and functional experiments showed that FEN1 knockdown could inhibit cell proliferation by inducing cell cycle arrest from G2 to M phase. As an important cell cycle regulatory protein, Cdc25C is involved in activating the Cyclin B1/CDK1 complex in cells to initiate mitosis 27. The Cyclin B1/CDK1 complex is a key regulator of G2/M transition 28. The Cyclin B1/CDK1 complex can phosphorylate varieties of proteins prior to G2/M transition, which starts the mitotic events, including nuclear envelope breakdown, centrosome separation and chromosome condensation 29. In this study, we found that FEN1 expression was positively correlated with the expression levels of Cdc25C, CDK1 and Cyclin B1. Moreover, the results given by the bioinformatics tool (GEPIA) came to the same conclusion. However, our studies only investigated the correlation between FEN1 and Cdc25C, CDK1 or Cyclin B1, and the further mechanism remains to be explored.

In summary, our study suggests that FEN1 promotes the proliferation, migration and invasion of HCC cells by activating cell cycle transition from G2 to M phase though modulating Cdc25C, CDK1 and Cyclin B1 expressions. FEN1 is an important biomarker for predicting the prognosis of HCC patients. Our findings may provide a new focus in the search for treatment strategies for liver cancer.

## Supplementary Material

Supplementary figures and tables.

## Figures and Tables

**Table 1:** FEN1 expression in HCC and clinicopathological characteristics (n =57).

FEN1 expression			
	Low(n=22)	High(n=35)	P value
Gender			1.000
Male	17	28	
Female	5	7	
Age (years)			0.059
≤ 65	16	33	
>65	6	2	
Tumor grade			0.011
Well	4	2	
Moderate	17	21	
Poor	1	12	
Tumor T stage			0.006
T1	15	8	
T2	4	12	
T3	2	8	
T4	1	7	
Tumor N stage			1.000
N0	20	32	
N1	2	3	
Tumor M stage			0.043
M0	22	27	
M1	0	8	
Tumor stage			0.006
Ⅰ	15	8	
Ⅱ	4	12	
Ⅲ	1	7	
Ⅳ	2	8	

## References

[1] Sung H, Ferlay J, Siegel RL, Laversanne M, Soerjomataram I, Jemal A (2021). Global Cancer Statistics 2020: GLOBOCAN Estimates of Incidence and Mortality Worldwide for 36 Cancers in 185 Countries. CA Cancer J Clin.

[2] Llovet JM, Kelley RK, Villanueva A, Singal AG, Pikarsky E, Roayaie S (2021). Hepatocellular carcinoma. Nat Rev Dis Primers.

[3] Wang R, Liu Y, Sun H, Wang T, Li C, Fan J (2021). Estradiol is significantly associated with prognosis in non-surgical liver cancer patients: from bench to bedside. Aging (Albany NY).

[4] Vogel A, Meyer T, Sapisochin G, Salem R, Saborowski A (2022). Hepatocellular carcinoma. Lancet.

[5] Balakrishnan L, Bambara RA (2013). Flap endonuclease 1. Annual review of biochemistry.

[6] Mengwasser KE, Adeyemi RO, Leng Y, Choi MY, Clairmont C, D'Andrea AD (2019). Genetic Screens Reveal FEN1 and APEX2 as BRCA2 Synthetic Lethal Targets. Mol Cell.

[7] Xu X, Shi R, Zheng L, Guo Z, Wang L, Zhou M (2018). SUMO-1 modification of FEN1 facilitates its interaction with Rad9-Rad1-Hus1 to counteract DNA replication stress. Journal of molecular cell biology.

[8] Zhang J, Xie S, Zhu JK, Gong Z (2016). Requirement for flap endonuclease 1 (FEN1) to maintain genomic stability and transcriptional gene silencing in Arabidopsis. The Plant journal: for cell and molecular biology.

[9] Algasaier SI, Finger LD, Bennet IA, Grasby JA (2018). Flap Endonuclease 1 Mutations A159V and E160D Cause Genomic Instability by Slowing Reaction on Double-Flap Substrates. Biochemistry.

[10] Liu L, Zhou C, Zhou L, Peng L, Li D, Zhang X (2012). Functional FEN1 genetic variants contribute to risk of hepatocellular carcinoma, esophageal cancer, gastric cancer and colorectal cancer. Carcinogenesis.

[11] Sato M, Girard L, Sekine I, Sunaga N, Ramirez RD, Kamibayashi C (2003). Increased expression and no mutation of the Flap endonuclease (FEN1) gene in human lung cancer. Oncogene.

[12] Zeng X, Qu X, Zhao C, Xu L, Hou K, Liu Y (2019). FEN1 mediates miR-200a methylation and promotes breast cancer cell growth via MET and EGFR signaling. Faseb j.

[13] Li C, Zhou D, Hong H, Yang S, Zhang L, Li S (2019). TGFβ1- miR-140-5p axis mediated up-regulation of Flap Endonuclease 1 promotes epithelial-mesenchymal transition in hepatocellular carcinoma. Aging (Albany NY).

[14] Pu J, Wang J, Qin Z, Wang A, Zhang Y, Wu X (2020). IGF2BP2 Promotes Liver Cancer Growth Through an m6A-FEN1-Dependent Mechanism. Front Oncol.

[15] Bian S, Ni W, Zhu M, Zhang X, Qiang Y, Zhang J (2022). Flap endonuclease 1 Facilitated Hepatocellular Carcinoma Progression by Enhancing USP7/MDM2-mediated P53 Inactivation. Int J Biol Sci.

[16] Rumgay H, Arnold M, Ferlay J, Lesi O, Cabasag CJ, Vignat J (2022). Global burden of primary liver cancer in 2020 and predictions to 2040. J Hepatol.

[17] Siegel RL, Miller KD, Wagle NS, Jemal A (2023). Cancer statistics, 2023. CA Cancer J Clin.

[18] Sia D, Villanueva A, Friedman SL, Llovet JM (2017). Liver Cancer Cell of Origin, Molecular Class, and Effects on Patient Prognosis. Gastroenterology.

[19] Nagaraju GP, Dariya B, Kasa P, Peela S, El-Rayes BF (2022). Epigenetics in hepatocellular carcinoma. Seminars in cancer biology.

[20] Parikh ND, Pillai A (2021). Recent Advances in Hepatocellular Carcinoma Treatment. Clin Gastroenterol Hepatol.

[21] Huang A, Yang XR, Chung WY, Dennison AR, Zhou J (2020). Targeted therapy for hepatocellular carcinoma. Signal Transduct Target Ther.

[22] Guo E, Ishii Y, Mueller J, Srivatsan A, Gahman T, Putnam CD (2020). FEN1 endonuclease as a therapeutic target for human cancers with defects in homologous recombination. Proc Natl Acad Sci U S A.

[23] Coffman JA (2004). Cell cycle development. Developmental cell.

[24] Suski JM, Braun M, Strmiska V, Sicinski P (2021). Targeting cell-cycle machinery in cancer. Cancer Cell.

[25] Murray A (1994). Cell cycle checkpoints. Current opinion in cell biology.

[26] Matthews HK, Bertoli C, de Bruin RAM (2022). Cell cycle control in cancer. Nature reviews Molecular cell biology.

[27] Sisinni L, Maddalena F, Condelli V, Pannone G, Simeon V, Li Bergolis V (2017). TRAP1 controls cell cycle G2-M transition through the regulation of CDK1 and MAD2 expression/ubiquitination. J Pathol.

[28] Nigg EA (2001). Mitotic kinases as regulators of cell division and its checkpoints. Nature reviews Molecular cell biology.

[29] Wang Z, Fan M, Candas D, Zhang TQ, Qin L, Eldridge A (2014). Cyclin B1/Cdk1 coordinates mitochondrial respiration for cell-cycle G2/M progression. Developmental cell.

