# Supplementary material for: FEN1 Promotes Hepatocellular Carcinoma Progression by Activating Cell Cycle Transition from G2 To M Phase
Source: J Cancer. 2024 Jan 1;15(4):981–9. doi: 10.7150/jca.88160 (PMC10788719; doi:10.7150/jca.88160)
Supplement: Supplementary file 1 — Supplementary figures and tables. [file jcav15p0981s1.pdf]

Supplemental Figures

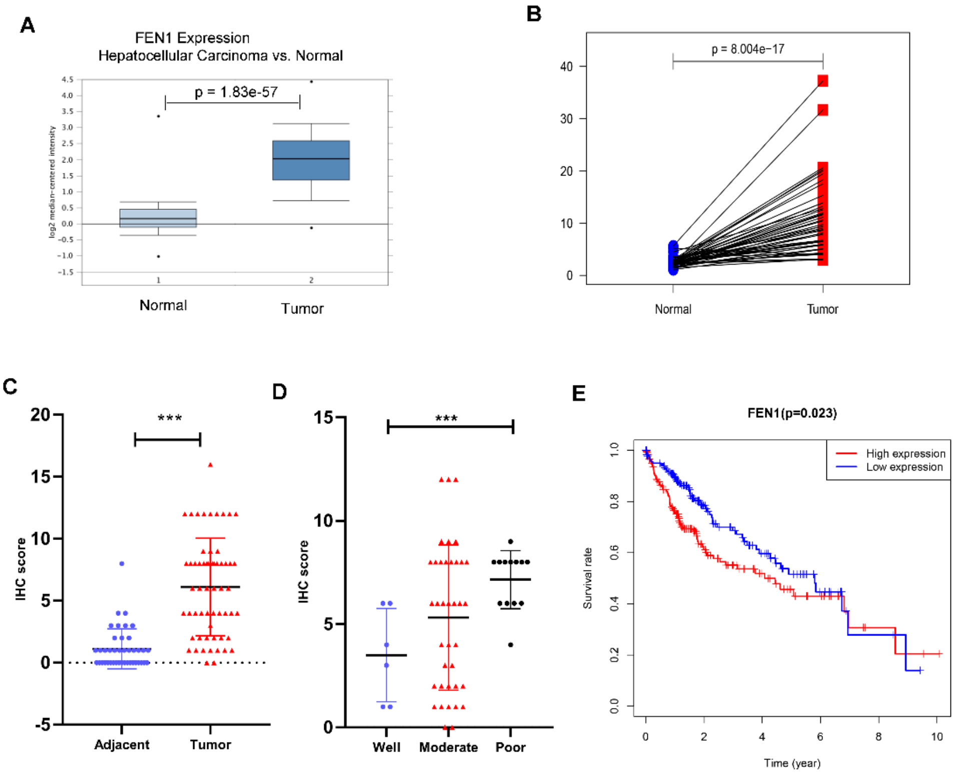

Figure S1. (A) FEN1 expression levels of HCC tissues and normal tissues in the Oncomine database. (B) Paired plot showing the difference in FEN1 expression between 50 pairs of HCC tissues and adjacent normal tissues from the TCGA database. (C) Protein levels of FEN1 in 57 pairs of HCC tissues and adjacent normal tissues according to IHC score. (D) High FEN1 expression was correlated with tumor grade. (E) Comparison between low and high FEN1-expression groups in the TCGA-LIHC cohort revealed that high FEN1 was associated with poor overall survival. *** $P < 0.001$.

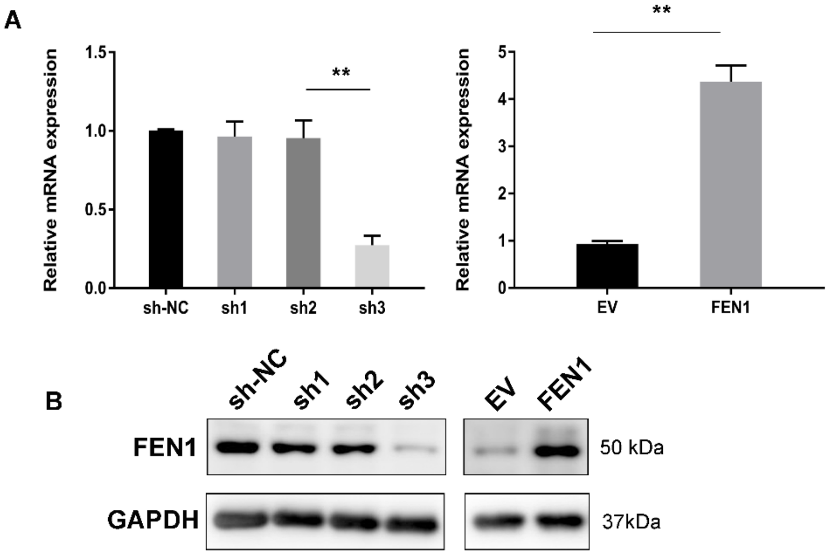

Figure S2. (A) RT-qPCR analysis of FEN1 expression after lentiviral expression of sh-FEN1 and FEN1-overexpressing. (B) Western blot detection of FEN1 expression after lentiviral expression of sh-FEN1 and FEN1-overexpressing. ** $P < 0.01$.

Supplemental Tables

Table S1. Primer sequences for RT-qPCR assays

	Primers	Sequence (5' to 3')
1	GAPDH-F	TGACATCAAGAAGGTGGTGAAGCAG
2	GAPDH-R	GTGTCGCTGTTGAAGTCAGAGGAG
3	FEN1-F	TCATCCAGAAGCACAAGAGCATCG
4	FEN1-R	CCAGCACCTCAGGTTCCAAGAAG
5	Cyclin B1-F	GAGCTATCCTCATTGACTGG
6	Cyclin B1-R	CATCTTCTTGGGCACACAAC
7	Cdc25C-F	AGAAGCCCATCGTCCCTTTGGA
8	Cdc25C-R	GCAGGATACTGGTTCAGAGACC
9	CDK1-F	GGAAACCAGGAAGCCTAGCATC
10	CDK1-R	GGATGATTCAGTGCCATTTTGCC

Table S2. Sequences of shRNAs for FEN1 gene

	shRNAs	Sequence
1	FEN1-shRNA1	TCAAGTTCATGTGTGGTGA
2	FEN1-shRNA2	CGCCTCGATGATTTCTTCA
3	FEN1-shRNA3	AGGCAGTGACTACTGTGAG

Table S3. Clinical characteristics comparison between Own cohort and TCGA cohort			
	Own Cohort(n=57)	TCGA Cohort(n=377)	P value
Gender (%)			0.117
Male	45 (78.9)	255 (67.6)	
Female	12 (21.1)	122 (32.4)	
Age (years) (%)			0.001
≤ 65	49 (86.0)	235 (62.5)	
> 65	8 (14.0)	141 (37.5)	
Tumor T stage (%)			0.019
T1	31 (54.4)	185 (49.3)	
T2	13 (22.8)	95 (25.3)	
T3	6 (10.5)	81 (21.6)	
T4	7 (12.3)	13 (3.5)	
TX	0 (0.0)	1 (0.3)	
Tumor N stage (%)			<0.001
N0	50 (87.7)	257 (68.4)	
N1	7 (12.3)	4 (1.1)	
NX	0 (0.0)	115 (30.6)	
Tumor M stage (%)			<0.001
M0	49 (86.0)	272 (72.1)	
M1	8 (14.0)	4 (1.1)	
MX	0 (0.0)	101 (26.8)	
Tumor stage (%)			<0.001
I	23 (40.4)	175 (49.6)	
II	20 (35.1)	87 (24.6)	
III	5 (8.8)	86 (24.4)	
IV	9 (15.8)	5 (1.4)	
